# Aerosol generation during general anesthesia is comparable to coughing: An observational clinical study

**DOI:** 10.1111/aas.14022

**Published:** 2022-01-11

**Authors:** Lotta‐Maria Oksanen, Enni Sanmark, Svetlana Sofieva, Noora Rantanen, Mari Lahelma, Veli‐Jukka Anttila, Lasse Lehtonen, Nina Atanasova, Eero Pesonen, Ahmed Geneid, Antti‐Pekka Hyvärinen

**Affiliations:** ^1^ 3835 Faculty of Medicine University of Helsinki Helsinki Finland; ^2^ 60655 Department of Otorhinolaryngology and Phoniatrics—Head and Neck Surgery Helsinki University Hospital Helsinki Finland; ^3^ 3835 Faculty of Biological and Environmental Sciences Molecular and Integrative Biosciences Research Programme University of Helsinki Helsinki Finland; ^4^ 3835 Faculty of Science, Mathematics and Statistics University of Helsinki Helsinki Finland; ^5^ 60655 HUS Inflammation Centre Helsinki University Hospital Helsinki Finland; ^6^ 60655 HUS Diagnostic Centre HUSLAB Helsinki University Hospital Helsinki Finland; ^7^ Finnish Meteorological Institute Helsinki Finland; ^8^ 60655 Department of Anesthesiology, Intensive Care and Pain Medicine Helsinki University Hospital Helsinki Finland

**Keywords:** aerosol, airborne transmission, anesthesia, COVID‐19, extubation, intubation, mask ventilation

## Abstract

**Background:**

Intubation, laryngoscopy, and extubation are considered highly aerosol‐generating procedures, and additional safety protocols are used during COVID‐19 pandemic in these procedures. However, previous studies are mainly experimental and have neither analyzed staff exposure to aerosol generation in the real‐life operating room environment nor compared the exposure to aerosol concentrations generated during normal patient care. To assess operational staff exposure to potentially infectious particle generation during general anesthesia, we measured particle concentration and size distribution with patients undergoing surgery with Optical Particle Sizer.

**Methods:**

A single‐center observative multidisciplinary clinical study in Helsinki University Hospital with 39 adult patients who underwent general anesthesia with tracheal intubation. Mean particle concentrations during different anesthesia procedures were statistically compared with cough control data collected from 37 volunteers to assess the differences in particle generation.

**Results:**

This study measured 25 preoxygenations, 30 mask ventilations, 28 intubations, and 24 extubations. The highest total aerosol concentration of 1153 particles (p)/cm³ was observed during mask ventilation. Preoxygenations, mask ventilations, and extubations as well as uncomplicated intubations generated mean aerosol concentrations statistically comparable to coughing. It is noteworthy that difficult intubation generated significantly fewer aerosols than either uncomplicated intubation (*p* = .007) or coughing (*p* = 0.006).

**Conclusions:**

Anesthesia induction generates mainly small (<1 µm) aerosol particles. Based on our results, general anesthesia procedures are not highly aerosol‐generating compared with coughing. Thus, their definition as high‐risk aerosol‐generating procedures should be re‐evaluated due to comparable exposures during normal patient care.

**Implication Statement:**

The list of aerosol‐generating procedures guides the use of protective equipments in hospitals. Intubation is listed as a high‐risk aerosol‐generating procedure, however, aerosol generation has not been measured thoroughly. We measured aerosol generation during general anesthesia. None of the general anesthesia procedures generated statistically more aerosols than coughing and thus should not be considered as higher risk compared to normal respiratory activities.

**Editorial Comment**

Induction of general anesthesia, with positive pressure delivery of gas to the lungs and intubation, has been considered a high‐risk procedure for aerosol generation with relevance for aerosol‐transmitted disease. How much aerosol generation that occurs in this context is compared in this study with simple coughing. Anesthesia airway and noninvasive ventilation does not appear to generate more aerosol than usual respiratory activities.

## 1 INTRODUCTION

Airborne transmission of infectious viruses has been intensively researched during the COVID‐19 pandemic. Currently, there is a raising awareness of aerosol transmission as an important, even predominant route over both long and short distances.^1^, ^2^, ^3^, ^4^, ^5^ Breathing and talking produce fine aerosols that have been shown to carry SARS‐CoV‐2 RNA copies in the absence of aerosol‐generating procedures (AGPs).^6^, ^7^ However, use of the personal protective equipment (PPE) that protects from airborne infection has been recommended mainly during assumed AGPs in anesthesia and surgery.^8^, ^9^ AGPs are medical procedures that are thought to generate high number of aerosols causing an increased risk for respiratory pathogen transmission. However, there is no consensus of which procedures are significantly aerosol generating and currently Centers for Disease Control and Prevention (CDC) states: “*There is neither expert consensus, nor sufficient supporting data, to create a definitive and comprehensive list of AGPs for healthcare settings.”* ^10^

The list of AGPs varies by country, organization and even by medical specialty. World Health Organization (WHO) lists currently the following procedures as AGPs: tracheal intubation, non‐invasive ventilation, manual ventilation before intubation, tracheotomy, bronchoscopy, cardiopulmonary resuscitation, sputum induction, autopsy, and dentistry procedures.^11^ Procedures have been raised to AGP‐listings based on mainly case‐control and retrospective cohort studies.^12^, ^13^, ^14^, ^15^, ^16^, ^17^ In a systematic review, the quality of the existing evidence regarding AGPs was estimated to be low.^18^ It is necessary to determine the level of significant aerosol generation to define an AGP. To date, there is no exact quantified definition of an AGP. In general, AGPs are considered to produce more aerosol than coughing, which has been regarded as a reference for AGP in earlier studies.^19^, ^20^, ^21^ A paradigm shift is currently under discussion: to justify the use of a higher level of PPE in AGP classified medical procedure compared to normal patient contact, the AGP should generate aerosol concentrations that are higher than in normal patient care where caregivers are constantly exposed for the particles generated by breathing, speaking, and coughing.^22^ As coughing is known to generate higher aerosol amounts than other regular respiratory activities,^23^ we considered it similarly to previous statements as a justified benchmark that generates an upperlimit for everyday clinical aerosol exposure.

Currently, multiple countermeasures have been adopted for the surgeries due to the expected aerosol generation during general anesthesia and especially intubation. The number of staff members working in the operating room (OR) has been minimized, and aerosol boxes and other novel devices have been developed to improve healthcare workers’ (HCWs) safety.^24^, ^25^ However, these countermeasures have led to negative effects on interactions of the operating team and the quality of surgery.^26^ Thus, there is a clear need to define if they are necessary. To the best of our knowledge, only two prospective studies on aerosol production in tracheal intubation and extubation with a small number of patients have been published.^19^, ^27^ Results with larger numbers of patients, clear particle size distributions during different anesthesia procedures and comparisons with reference data collected from multiple persons are still needed.

This study aims to analyze the operational staff’s exposure to occurrence and amount of aerosol generation in the OR environment during all steps in general anesthesia: preoxygenation, non‐invasive mask ventilation, intubation, and extubation. Real‐time quantitative measurements of the generation of aerosol particles during airway managing procedures are critical to understanding the level of risk during surgeries, guiding PPE usage, and minimizing unnecessary changes in operating protocols while ensuring the safety of HCWs.

## 2 METHODS

### 2.1 Ethical considerations

All procedures involving human participants were conducted in accordance with the ethical standards of the institutional research committee and the 1964 Declaration of Helsinki and its later amendments. The Ethics Committee of Helsinki University Hospital (PL 705, 00029 HUS Biomedicum Helsinki 2 C 7th floor, Tukholmankatu 8 C, Helsinki. Chairperson Markus Perola) approved the study protocol 29 May 2020 (HUS/1701/2020). All patients provided written informed consent prior to their participation.

### 2.2 Patients

Aerosol monitoring was conducted in Helsinki University Hospital (HUS) between August and October 2020 during general anesthesia ENT operations in ORs. Thirty‐nine adult patients scheduled for surgery under general anesthesia with endotracheal intubation were included in the study. Patients with tracheostomy, airway anomaly, or acute COVID‐19 infection were excluded.

Reference coughing data were collected from 37 healthy volunteers from a total of 252 coughs between December 2020 and February 2021 in HUS. Exactly similar collection methodology and same Ors with the same ventilation systems were used in both this study and for reference data. No additional collection methods, for example, funnels, were used as we wanted to measure the exposure for the aerosol particles in a certain spot to reflect the exposure for the staff member during the operation instead of overall particle generation. Coughing data were published in our other study.^28^ Volunteers did not have any signs of an acute respiratory infection during measurement.

No previous data to perform a valid power‐calculation exist for the studied anesthesia procedures, as similar measurements on these are very scarce. Thus, we measured as many anesthesia inductions as possible within the limits of the availability of the optical particle sizer and the feasibility to obtain data during the studied period. Because there were limitations in the availability of the OPS we were not able to measure all anesthesia procedures from all studied patients causing smaller n for studied categories than overall *n*.

### 2.3 Particle measurement

All measurements were conducted in ORs with laminar flow and high air change rate per hour (ACH) prospectively, in cooperation with the operating staff without any change to the arrangements of the room ventilation, instruments, personnel, or equipment.

Particle number and size distributions in the size range from 0.3 to 10 µm were measured with an Optical Particle Sizer (OPS) (TSI model 3330). The OPS is based on the principle of optical light scattering from single particles. While the number of pulses directly yields the concentration of particles, the OPS reports the optical size of these particles in 16 size bins every 10 s. These size bins have been factory‐calibrated with polystyrene latex (PSL) particles having a refractive index of 1.59. The OPS measures continuously single particle detection. Thus, catching the actual particles of short‐lived events like coughs are not dependent on the resolution used by the instrument. However, a choice has to be made considering the smearing in (1) temporal resolution and (2) counting statistics, as a shorter resolution increases random noise in the data. 10 s was found to be a good compromise. The OPS data were used without any further corrections to size.

To assure data quality, the OPS was factory‐calibrated before the measurements. The nominal flow rate of 1 l/min of the instrument was checked regularly with a mass flow meter (TSI model 4143). The flow rate varied by ±2% during the whole measurement period. In addition, the sizing of the instrument was checked periodically with 900 nm PSL particles, and the concentration was compared against another OPS unit.

The study intended to measure particles that remain airborne but are large enough to carry pathogens. Previous studies have shown that pathogens predominate in aerosol particles <5 µm.^29^, ^30^, ^31^ In our study, the measured particles were categorized as <1 µm, 1–5 µm and >5 µm for data analyses. The OPS collects particles actively at the point of the inlet. Therefore it describes the particle concentration at that point location of the particle field. Thus it simulates well the exposure of the operating staff, who can also be considered to “collect aerosols” at a given point location. The OPS was placed on the side of the bed adjacent to the patient’s head to reflect the distance of operation staff; preferably imitating the distance of the anesthesiologist’s airways from the patient’s airways (Table 1). However, in a real‐life situation, the position of the OPS is highly dependent on the feasibility to conduct the measurement without intervening with an operator or operating equipment, thus the OPS was placed as close as possible within these limits. The cough measurements used in the comparison were also measured from several distances, respectively.

**TABLE 1:** Distance of OPS from patient during studied procedures

	Mean (cm)	Range (cm)
Preoxygenation with mask (*n* = 24)	136	40–210
Mask ventilation (*n* = 29)	131	40–210
Intubation (*n* = 28)	116	40–210
Extubation (*n* = 24)	116	40–180

Note

Missing values in preoxygenation with mask (*n* = 1) and mask ventilation (*n* = 1).

### 2.4 Measurement protocol

Airway management during anesthesia inductions followed the same schema: preoxygenation (from the start of oxygen supply to start of mask ventilation), non‐invasive mask ventilation (from the start of mask ventilation to removal of the mask from patient’s face), direct laryngoscopy and tracheal intubation (including inflation of intubation tube cuff/s and securing of intubation tube by taping). Extubation was defined to start when the cuff was emptied and to end when the tube was removed. No aerosolized lidocaine was used during intubations. Pharma Systems mini port 6120PS, which filters >99.99% of particles and pathogens, was used to filter exhaled air. A similar filter was used in the ventilator.

A research nurse followed all operations in the OR. She registered beginnings and ends of procedures, used equipment, staff movement, number of intubation attempts and patients’ reactions (cough, movement, etc.) during both intubation and extubation. Possible disturbances in the OR that could affect the data, for example use of other instruments simultaneously, different posture for intubation or door opening, were marked and these measurement points were excluded. Also, intubations and extubations in which the exact end‐point was not clear in the data markings were excluded (*n* = 6 intubations, *n* = 1 extubation). All staff used RII surgical masks, which markedly reduce aerosol release, as seen in many earlier studies.^32^, ^33^ Intubations were categorized as normal (one attempt) and difficult (more than one attempt). Extubations were categorized for coughing and non‐coughing and regarding the tube used (normal vs. laser). Additionally, the relations of age and BMI were evaluated as explaining factors for differences in mask fitting.

### 2.5 Measured references

All procedures were compared with coughing data of 37 healthy volunteers to determine whether aerosol generation was higher than expectable exposures during normal patient care.^28^ All reference measurements were done using an identical methodology and the same particle sizer in the same Ors. Exact particle numbers from measured references are seen in Table S1.

### 2.6 Ventilation information in operating rooms

The background aerosol size distributions were measured separately for each OR. The air change rate varied in the different Ors between 30.23 and 60.67 ACH, which is above the American Institute of Architects (AIA) and UK guidelines of a minimum of 25 ACH.^34^, ^35^ The operating rooms had Recair 4C or INPO‐1.5 ventilation systems with HEPA‐14 filtration, and ultra‐clean ventilation in the laminar area in the central area of the OR of 1176 l/s–1478 l/s generating 363.35–572.83 ACH to the laminar area. The relative humidity varied between 25.1% and 75.3%. OR humidity was highest in August 2020 and decreased as the outside temperature decreased. During operations, 80% of air was re‐circulated.

### 2.7 Data analysis

To present the data, concentrations of aerosol particles in discrete size ranges, *N_Dp1‐Dp2_* [p/cm^3^], were determined. In addition, particle size distributions were normalized by dividing the concentrations in each measurement size bin with the logarithm of the respective bin width, *dN/dlogDp* [p/cm^3^]. This enables presenting particle size distributions independent of the bin widths. For individual procedures, mean concentrations and size distributions with standard deviations were calculated. The mean was chosen as a statistical representative parameter, as it describes the average exposure dose during an individual procedure, therefore reflecting the associated infection risk.^36^

Statistical calculations were performed by using Microsoft Excel 2016 (Microsoft Corporation, Redmont, WA, USA), GraphPad Prism version 9.0.2 for Mac (GraphPad Software, San Diego, CA, USA) and R‐Studio version 1.3.959 (R Foundation for Statistical Computing, Vienna, Austria). Particle concentrations between individual procedures were observed to be log‐normally distributed, as previously reported.^19^ Parametric tests were used after logarithmic transformation. Measured particle concentrations generated during general anesthesia were compared with background data using paired (OR specific) one‐tailed t‐test and with cough data using unpaired two‐tailed t‐test in each discreet size range separately with Bonferroni correction of four consecutive procedures. A one‐tailed *t*‐test was used when compared with background data since all particle generation is expected to exceed low background particle concentration. Intubation types (normal vs. difficult), intubation tubes (normal vs. laser), and extubation types (cough vs. no cough) were tested with unpaired *t*‐test. Bivariate correlations were tested with Pearson’s correlation test. *p*‐values < .05 were considered significant, except for *p* < .0125 after Bonferroni correction of four consecutive procedures. The manuscript was written in accordance with the STROBE principles.

## 3 RESULTS

### 3.1 Patients

Anesthesia procedures were measured for 39 patients (56% men, 44% women). The median age of all measured patients was 55 (range 19–85) years, and the mean BMI was 26.7 (range 15.6–44.9) kg/m².

### 3.2 Background aerosol concentration

Very low background concentrations (maximum mean concentration 0.017 particles/cm³) allowed accurate evaluation of the particles generated during the procedures. The total particle concentration for the background was 0.005 ± 0.018 and measured a maximum 0.228 particles/cm^3^. Notably, the lower limit of standard deviation in all measured procedures was 0.000 particles/cm³, as the very clean measurement environment and laminar ventilation produced multiple measure points with zero detectable particles. All procedures were statistically aerosol‐generating compared to the background and produced significantly particles in all size categories (*p* ≤ .001).

### 3.3 Particle concentrations and size distributions

Measured parts can be divided into four main procedures: preoxygenation, mask ventilation, intubation, and extubation. The median duration of the procedures was 1 min 50 s (IQR ±1 min 20 s) min for preoxygenation, 2 min 40 s (IQR ±1 min 10 s) min for mask ventilation, 3.00 min (IQR ±1 min 40 s) min for intubation and 20 s (IQR ±35 s) min for extubation. Particle generation compared with coughing is described in detail in Table S1.

All procedures were comparable statistically to concentrations seen in coughing in total particle concentration (*p* = .224–.870) and in all discreet size ranges (<1 µm: *p* = .220–0.883; 1–5 µm *p* = .051–.225; >5 µm: *p* = .020–.319) after Bonferroni correction. Mask ventilation generated the highest measured individual total particle concentration of 1154 particles/cm³. Aerosol generation during mask ventilation varied greatly (range of the total particle concentration means during mask ventilation was 0.000–94.919 particles/cm³). We evaluated the possible relations of age and BMI with mask fitting. Correlations were not statistically significant between total particle concentration means and age (rₛ = 0.041, *p* = .828) or BMI (rₛ = −0.047, *p* = .801).

**FIGURE 1:**
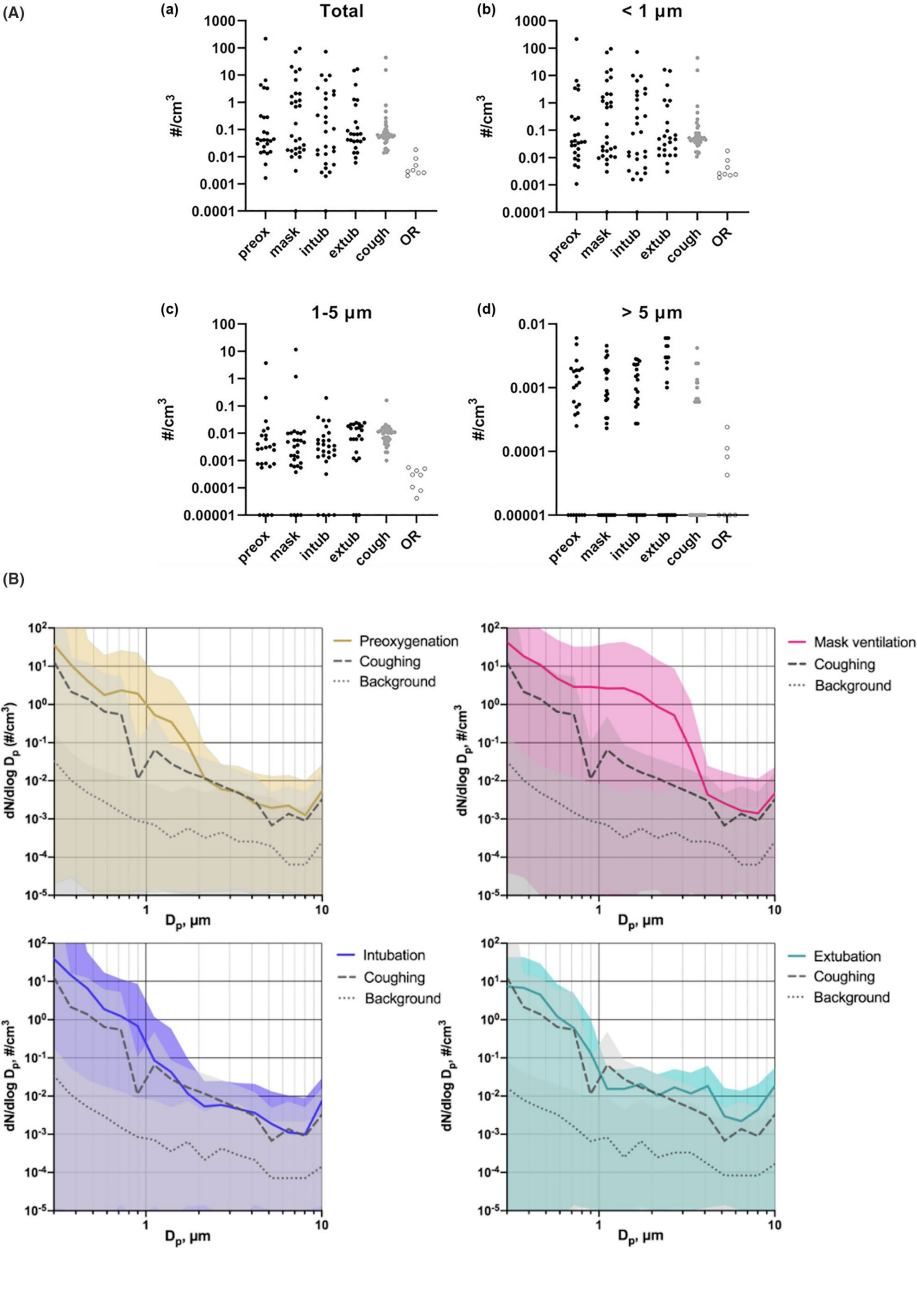
Particle concentration (A) and size distribution (B) of preoxygenation, mask ventilation, intubation and extubation compared to cough controls and operation room background. (A) Total particle concentration (A), particles < 1 μm (B), particles 1–5 μm (C) and particles > 5 μm. (D) during consecutive procedures: Preoxygenation (preox), mask ventilation (mask), intubation (intub) and extubation (extub) in black dots. Cough controls as grey dots and operation room background (OR) as circles. OR background was statistically significantly lower than any anesthesia procedure in all particle sizes (all *p* ≤ .001). Note the logarithmic scale on the y‐axis. To be depicted, the zero values have been replaced with the lowest value of the y‐axis. (B) Average size distribution of observed aerosols and average fractions of these aerosols in different size ranges compared with background and coughing data in four main cathegories (preoxygenation, yellow; mask ventilation, red; intubation, blue; and extubation, green) expressed as mean ± standard deviation. Dp refers to diameters of the observed particles and dN/dlogDp is the concentration expressed as particles per cubic centimetre

Figure 1 shows particle concentrations (A) and particle size distributions (B) in different anesthesia procedures.

**FIGURE 2:**
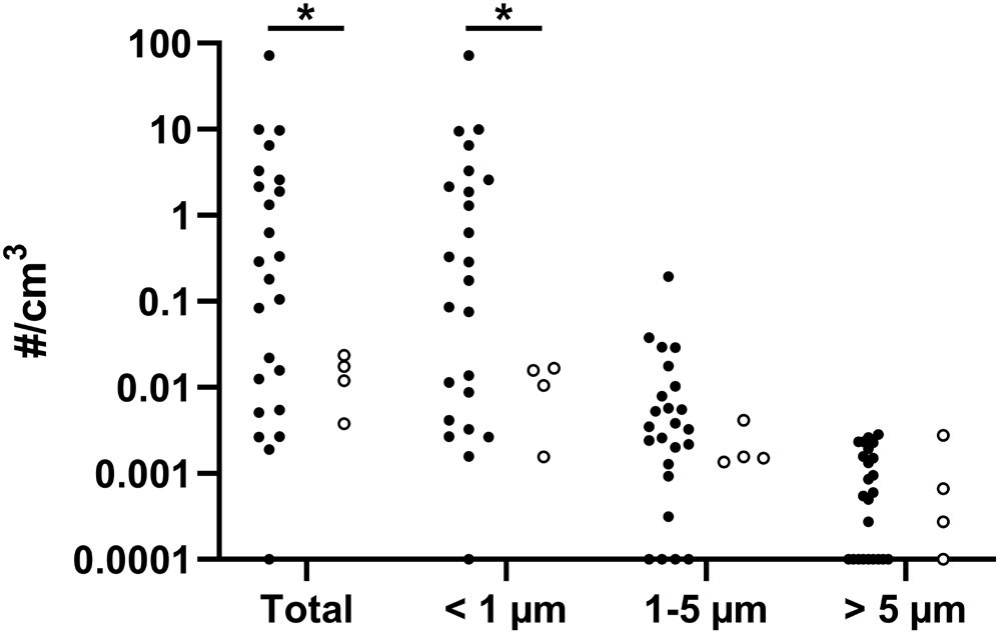
Particle concentration with normal and difficult intubation. Total particle concentration and particle concentrations in discreet size ranges during intubation in patients with normal (black dots, *N* = 24) and difficult (circles, *N* = 4) intubation. Potential mask ventilation between intubation attempts was excluded from the analysis. *p ≤ .001, normal vs. difficult intubation. Note the logarithmic scale on the y‐axis. To be depicted, the zero values have been replaced with the lowest value of the y‐axis

Intubation and extubation were further divided into subcategories. Difficult intubation produced significantly less particles than normal intubation (*p* ≤ .01) or coughing (*p* = .006). Mean detected particle concentration during normal intubation was thus over 500 times higher than during difficult intubation (mean 7.080 vs. 0.013 particles/cm³). Particle concentration differences between normal and difficult intubations are seen in Figure 2. Potential mask ventilation between difficult intubation attempts was excluded from the analysis to assess the particle generation caused by the intubation.

**FIGURE 3:**
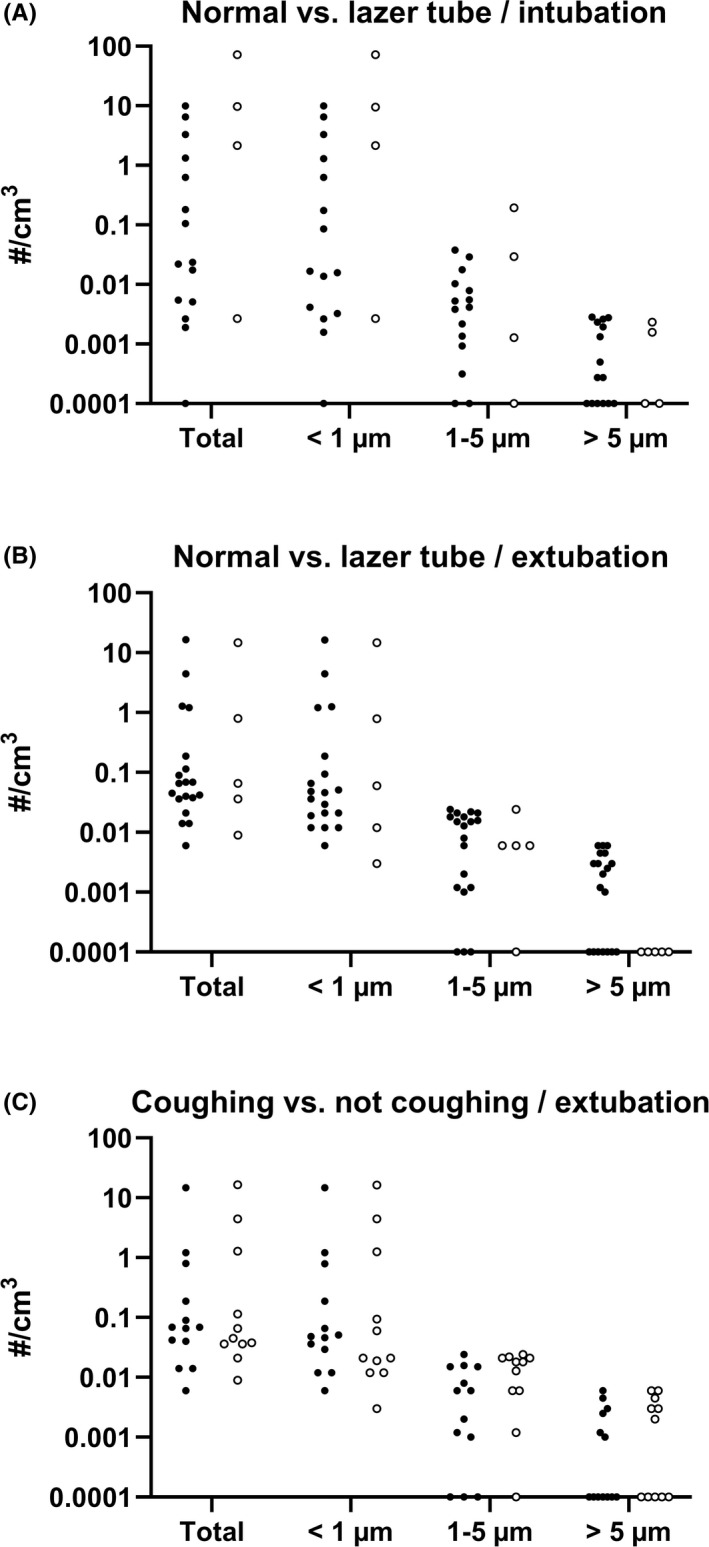
Particle concentration during intubation (A) and extubation (B) with normal and laser tube, and during extubation (C) with and without coughing. (A) Total particle concentration and particle concentrations in discreet size ranges during intubation in patients with normal (black dots) and laser (circles) intubation tube. (B) Total particle concentration and particle concentrations in discreet size ranges during extubation in patients with normal (black dots) and laser (circles) intubation tube. (C) Total particle concentration and particle concentrations in discreet size ranges during extubation in patients with (black dots) and without (circles) cough. Note the logarithmic scale on the y‐axis. To be depicted, the zero values have been replaced with the lowest value of the y‐axis

Extubations were further analyzed by intubation tube type used (normal or laser) and by whether patients were coughing or not coughing during extubation. No statistical differences emerged between these subgroups. Coughing occurred in 40% of extubations with a laser tube and in 47% of extubations with a normal tube. Detailed information is presented in Figure 3 and Table S2.

**FIGURE 4:**
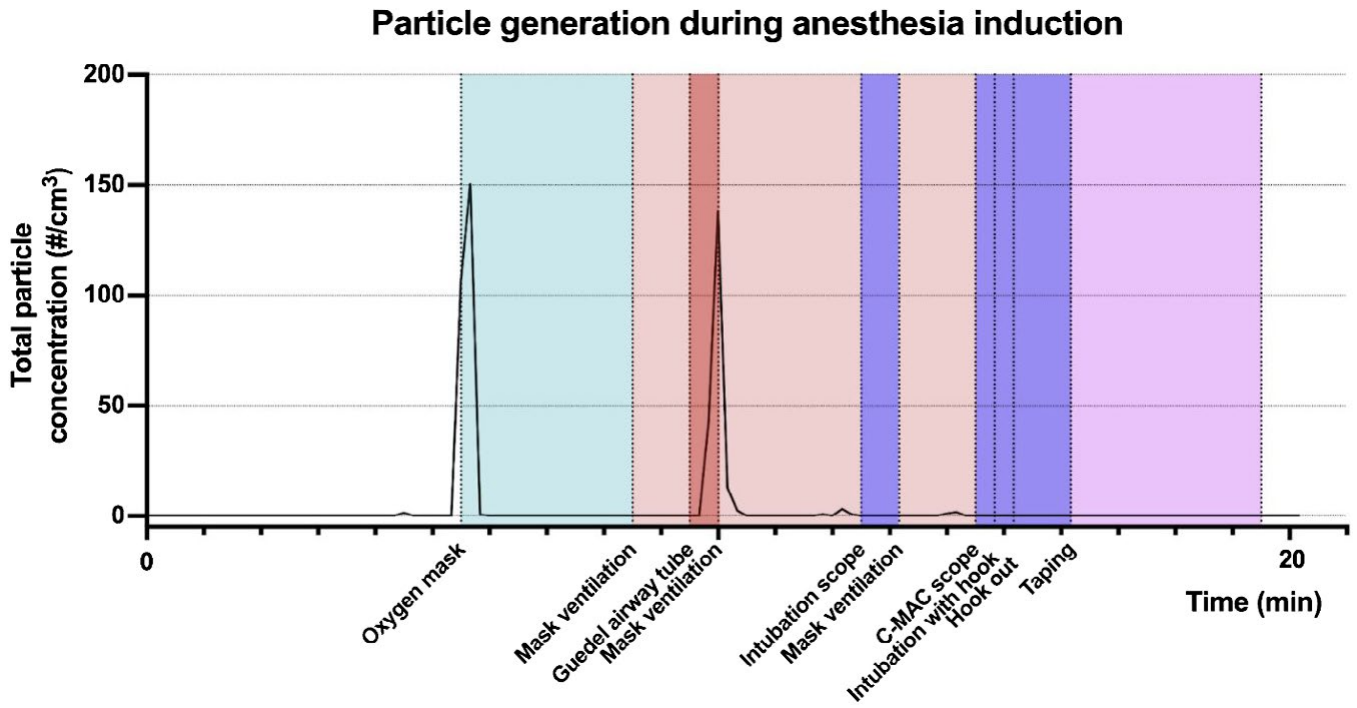
An example of total particle generation during anesthesia induction. Total particle generation during anaesthesia induction. An example from a difficult intubation. Particle concentration measured at 10‐s intervals. Periods of preoxygenation, mask ventilation, intubation and taping are colour‐coded, and starting points for the procedures are marked under the x‐axis. Operating room information: 58.5 m^2^, 140 m^3^, temp. 20.5°C, RH 37.5 %, room air exchanges 30 times/h. Particle generation is seen during mask ventilation due to face leakage. No aerosol generation is seen in any intubation tube insertion attempts. C‐MAC scope, videolaryngoscope. Green: preoxygenation with oxygen mask. Peach: mask ventilation. Red: insertion of the Gluedel airway tube. Blue: intubation (including direct/video laryngoscopy and tracheal intubation, inflation of intubation tube cuff/s and securing of intubation tube by taping). Pink: preparing for the operation

An example of measured particle generation during anesthesia induction is provided in Figure 4.

## 4 DISCUSSION

We measured aerosol generation and size distributions in different phases of general anesthesia, including preoxygenation, mask ventilation, intubation and extubation and their different variations under real‐life situations. None of the general anesthesia procedures generated a statistically significant higher amount of aerosols than coughing, and thus their definition as high‐risk AGPs should be re‐considered. Contrary to the previous assumption, difficult intubations generated significantly fewer aerosols than coughing and can rather be regarded as low‐risk AGP.

### 4.1 Preoxygenation and mask ventilation

Mean particle generation during preoxygenation and mask ventilation did not statistically exceed the mean aerosol generation of coughing, and thus listing these procedures as high‐risk AGPs is problematic. Still, aerosol generation was not significantly lower than coughing, either. The maximum concentrations were higher during preoxygenation and mask ventilation than ones measured during coughing, which may partly be explained by particles originating directly from the oxygen source.

This study observed that aerosol generation during bag‐valve‐mask ventilation varied greatly, from no detectable particles to the highest detected number of particles. Since all exhaust air was filtered and no leakage was detected from the bag‐valve‐mask, it is reasonable to expect that the particles observed during mask ventilation were released due to face‐mask seal leakage which has shown to be the most common leakage.^37^ Because of the many variants related to the fitting of the mask, small leakages quite often occur, as reflected by the results. In line with this, Dhillon et al.^27^ found that mask ventilation produced 200–300 times higher particle concentrations than the background. Furthermore, in small cohort‐studies, mask ventilation was associated with increased risk of SARS‐CoV‐1 transmission in health care workers.^14^, ^15^, ^16^ Thus, it is important to (1) use exhale valve filters to protect HCWs from airborne infections and (2) ensure as a tight fit as possible in the face‐mask interface. If ensuring tight fit is not possible, a higher aerosol exposure is expected and can be clinically significant especially when mask ventilation continues for several minutes.

In preoxygenation, most of the particles likely originate from the oxygen source and are potentially nonpathogenic. During mask ventilation, by contrast, air is directed into the patient’s airways by positive pressure. Air moistures as relative humidity rises and warms in the patient’s airways and aerosol particles will swell accordingly, growing in size and possibly collecting pathogens.^38^, ^39^ Particles that arise from the airways are more likely to be infective.

### 4.2 Intubation and extubation

During intubations, the mean particle generation did not exceed coughing statistically. Interestingly, difficult intubation produced significantly fewer aerosols than uncomplicated intubation. The higher amounts seen in normal intubations are possibly caused due to staff movement right after successful intubation often seen during taping. This potentially results in increased detection of particles not originating from the patient.

During difficult intubations, where the time spent for laryngoscopy and tube insertion increases, significantly fewer aerosols were generated than during coughing. This supports that intubation should be considered as low‐risk AGP. During intubation, the patient does not breathe and the movement of the tube is towards the lower airways. Thus, it is not surprising that intubation itself did not produce aerosols. The use of videolaryngoscopy can be regarded even safer procedure as it offers to the physician the possibility to stay further away from patients airway. However, there should not be other differences that should affect to aerosol generation between direct laryngoscopy and videolaryngoscopy.

Brown et al.^19^ observed similarly a negligible amount of aerosol production during intubation. By contrast, according to a systematic review including case‐control and cohort studies, tracheal intubation may be a risk factor for SARS‐CoV‐1 infection.^18^ Although intubation is associated with an increased risk of infections, other reasons than the actual laryngoscopy may be more significant for infection risk. We agree with Klompas et al.^40^ that the paradox that intubation is associated with higher infection risk is probably due to close range, for example, patients coughing and breathing heavily before intubation and given oxygen support, instead of intubation itself. We also agree with Wilson et al.^41^ that respiratory activities, such as coughing, can generate a higher risk for aerosol exposure than procedures classically classified as AGPs, as seen in our data during difficult intubations. Similarly, the exposure time during different respiratory activities and medical procedures should be considered as an important part when assessing the overall risk. Despite high aerosol amounts seen while coughing or in the peaks during mask ventilation, the overall cumulative exposure, and thus infective dose, may be considerably higher during for example long discussion with the patient.

In our study, extubation generated aerosol concentrations comparable to coughing. Instead, in Brown et al's. work extubation generated smaller amounts of aerosol than coughing.^19^ This difference potentially arrives from our larger cough data and thus variability seen between aerosol generation in different people who coughed. Extubation with coughing produced four times more particles (total mean 2.875 vs. 0.945 p/cm³) than non‐coughing even there was no statistically significant difference. This trend is similar than observed by Dhillon et al. who noted the highest peak increase during extubation when patient was coughing.^27^

### 4.3 Variability in aerosol generation

High variability is seen regarding both aerosol generation between different persons during coughing and in our measurements during general anesthesia procedures. This variability is consistent with previous studies and further research to explain the reasons is needed.^19^, ^27^, ^42^ This variation seen in respiratory activities including speaking and breathing has been speculated to explain also superspreading events.^43^ The precautionary principle should be applied to consider all patients to be potentially highly aerosol‐generating ones. This also results that high‐risk AGPs should exceed this natural variation to justify the use of better PPE. Regarding highly transmissible airborne pathogens the aerosol precautions should be adopted already in the regular patient care. Our results indicate rather that the mean aerosol concentrations in analyzed procedures are at the comparable level with coughing and upgrading PPE for these procedures alone is questionable considering the overall infection risk. However, an exception may be a situation where poorly sealed and possibly prolonged mask ventilation is expected in a potentially infectious patient.

In addition to the exposure threshold, determining the number of viral copies per average aerosol particle is essential for risk assessment regarding exposure to airborne pathogens. The size distribution of particles is important. SARS‐CoV‐2 has been found in multiple size ranges, but mainly detected viral copies have been in particles <5 µm.^6^, ^7^, ^44^ Overall, respiratory pathogens are found especially in particles <5 µm.^29^, ^30^, ^31^, ^45^ Most of the particles in our study were <1 µm of size. These small aerosols can remain in the air for long periods,^46^ and can be inhaled to the alveolar level,^47^ thus being a challenge in infection prevention. Still, it is not fully known whether aerosol concentration or the aerosol mass is more important regarding the infection risk. This research field is still in its infancy and more investigations are needed.

### 4.4 Strengths and weaknesses

This study analyzed aerosol generation during general anesthesia in normal OR environment assessing all main phases of airway management of anesthesia induction and extubation and explored the particle exposure that OR staff confronts during these procedures. We evaluated the results with similarly collected cough data, a generally accepted prerequisite to estimate the level of aerosol production compared to aerosol spikes seen in normal patien care. These results provide much‐needed information on currently listed AGPs in anesthesia and their measured particle generation.

Real‐time measurements in a clinical operating context are both a strength and a weakness of this study. The movement of the staff possibly increased the number of detected particles not originating from the patient. The movement was recorded by a research nurse, which helped the interpretation of the data. All raw data were quality controlled before analysis so that all measurement points outside the procedures being examined were removed from the analysis as well as points with possible interfering factors in the OR. This study method measures the particle exposure of OR staff members, it does not measure a complete number of all produced particles. The real life OR circumstances caused variation in the measurement distances, although still reflecting the exposure of the staff, this might underestimate the aerosol exposure in very near distance during studied procedures. However, as aerosols follow air flows, the aerosol concentration do not always follow linear decrease in the outside laboratory measurements. For studies to come, multiple similar measurement points from different distances at the same time is recommended to further understand the correlation between distance and environmental factors in ORs. It should be kept in mind that these measurements were conducted in highly ventilated ORs and if particles are seen in the OR it is expected that the cumulative number of particles is much higher in a conventional indoor environment and the clearance slower. However, the aerosol generation ratio is not affected by the change of the environment, thus enabling the expectation that the comparison between aerosol generation during coughing and studied procedures is generalizable to other environments. The interpretation of results becomes easier when we start to better understand the infectivity and spread of viruses, indicating that more multidisciplinary research is needed. Because of the quantitative nature of our data, we hope that these results can be further analyzed as general knowledge in this scientific field increases.

## 5 CONCLUSION

All procedures during general anesthesia generate mainly small, <1 µm, aerosol particles. These small aerosols can remain suspended in the air for long periods and can be inhaled to the alveolar level. According to our findings, the listing of preoxygenation, mask ventilation, intubation, and extubation as high‐risk AGPs should be re‐considered. Still, aerosol generation was comparable to coughing. Furthermore, due to the vast inter‐individual differences, with some patients high amounts of aerosol were observed. These results can be applied to the risk assessment of airborne infection in ORs.

## AUTHOR CONTRIBUTION

L‐MO and ES: Conceptualization, methodology, validation, formal analysis, investigation, resources, writing original draft, editing, project administration, funding. SS: Methodology, formal analysis, writing—original draft. NR: Formal analysis, visualization. ML: Methodology, formal analysis, visualization, writing—review & editing. V‐JA: Conceptualization, methodology, resources, writing—review & editing LL: Conceptualization, methodology, resources, writing—review & editing, funding. NA: Writing—review and editing, funding acquisition. EP: Methodology, formal analysis, visualization, writing—review & editing. AG: Project administration, conceptualization, writing—review & editing, supervision. A‐PH: Conceptualization, methodology, formal analysis, validation, writing original draft, editing.

## Supporting information

Table S1

Table S2
