# Supplementary material for: Aerosol generation during general anesthesia is comparable to coughing: An observational clinical study
Source: Acta Anaesthesiol Scand. 2022 Jan 11;66(4):463–72. doi: 10.1111/aas.14022 (PMC9303240; doi:10.1111/aas.14022)
Supplement: Supplementary file 1 — Table S1 [file AAS-66-463-s001.docx]

**Manuscript**: Aerosol generation during general anesthesia is comparable to coughing: an observational clinical study.

**Authors**: Oksanen et al.

**Supplemental Table 1**. Aerosol particle concentrations generated during anaesthesia procedures, and comparisons with particle concentrations of coughing references.

|  |  | N* | Mean ± SD | Max | Comparison  (p-values) |
| --- | --- | --- | --- | --- | --- |
|  |  |  |  |  | Coughing |
| Total particle concentration, particles/cm³ | Preoxygenation with mask | 25 | 5.464 ± 49.876 | 736.470 | 0.789 |
|  | Mask ventilation | 30 | 8.664 ± 65.709 | 1153.602 | 0.224 |
|  | Intubation | 28 | 6.155 ± 53.055 | 758.700 | 0.870 |
|  | Extubation | 24 | 1.978 ± 10.335 | 90.330 | 0.579 |
|  | *Backgroundᵃ* |  | *0.005 ± 0.018* | *0.228* |  |
|  | *Coughingᵇ* |  | *1.601 ± 13.772* | *195.528* |  |
| <1 𝜇m particle concentration, particles/cm³ | Preoxygenation with mask | 25 | 5.370 ± 49.137 | 724.770 | 0.883 |
|  | Mask ventilation | 30 | 7.852 ± 62.947 | 1153.602 | 0.220 |
|  | Intubation | 28 | 6.138 ± 53.030 | 758.694 | 0.863 |
|  | Extubation | 24 | 1.965 ± 10.331 | 90.270 | 0.798 |
|  | *Backgroundᵃ* |  | *0.005 ± 0.018* | *0.228* |  |
|  | *Coughingᵇ* |  | *1.588 ± 13.751* | *195.51* |  |
| 1-5 𝜇m particle concentration, particles/cm³ | Preoxygenation with mask | 25 | 0.093 ± 0.977 | 15.258 | 0.120 |
|  | Mask ventilation | 30 | 0.811 ± 12.201 | 208.434 | 0.056 |
|  | Intubation | 28 | 0.015 ± 0.164 | 3.534 | 0.051 |
|  | Extubation | 24 | 0.010 ± 0.014 | 0.078 | 0.225 |
|  | *Backgroundᵃ* |  | *0.000 ± 0.002* | *0.018* |  |
|  | *Coughingᵇ* |  | *0.012 ± 0.064* | *1.242* |  |
| >5 𝜇m particle concentration, particles/ cm³ | Preoxygenation with mask | 25 | 0.001 ± 0.003 | 0.018 | **0.020** |
|  | Mask ventilation | 30 | 0.001 ± 0.003 | 0.030 | 0.319 |
|  | Intubation | 28 | 0.001 ± 0.003 | 0.018 | 0.131 |
|  | Extubation | 24 | 0.003 ± 0.005 | 0.030 | 0.312 |
|  | *Backgroundᵃ* |  | *0.000 ± 0.001* | *0.006* |  |
|  | *Coughingᵇ* |  | *0.001 ± 0.002* | *0.012* |  |

Mean ± SD and maximum concentrations were calculated from all measured time points (every 10 s.) for each procedure. Measured minimum value in all anesthesia procedures and reference measurements in all size groups of particles was 0.000. *P*-values compared with coughing reference were calculated with two-tailed unpaired t-test. *P-*values <0.05 were considered statistically significant. *ᵃ* Background reference: Values presented as combination of all measured operation room background values (t-tests calculated from specific background for each anesthesia procedure). *ᵇ* Coughing reference. *Number of patients. SD, standard deviation.
