# Supplementary material for: Aerosol generation during general anesthesia is comparable to coughing: An observational clinical study
Source: Acta Anaesthesiol Scand. 2022 Jan 11;66(4):463–72. doi: 10.1111/aas.14022 (PMC9303240; doi:10.1111/aas.14022)
Supplement: Supplementary file 2 — Table S2 [file AAS-66-463-s002.docx]

**Manuscript**: Aerosol generation during general anesthesia is comparable to coughing: an observational clinical study.

**Authors**: Oksanen et al.

**Supplemental Table 2**. Aerosol particle concentrations generated during anesthesia procedure subcategories, and comparisons to particle concentrations of coughing references.

|  |  | N* | Mean ± SD | Max | Comparison  (p-values) |
| --- | --- | --- | --- | --- | --- |
|  |  |  |  |  | Coughing |
| Total particle concentration, particles/cm³ | Intubation, normal | 24 | 7.080 ± 56.863 | 758.700 | 0.556 |
|  | Intubation, difficult | 4 | 0.013 ± 0.010 | 0.054 | **0.006** |
|  | Extubation, normal tube | 19 | 1.792 ± 10.385 | 90.330 | 0.764 |
|  | Extubation, laser tube | 5 | 3.786 ± 10.327 | 29.334 | 0.588 |
|  | Extubation, non-coughing | 13 | 0.945 ± 4.651 | 29.334 | 0.799 |
|  | Extubation, coughing | 11 | 2.875 ± 13.463 | 90.330 | 0.577 |
|  | *Backgroundᵃ* |  | *0.005 ± 0.018* | *0.228* |  |
|  | *Coughingᵇ* |  | *1.601 ± 13.772* | *195.528* |  |
| <1𝜇m particle concentration, particles/cm³ | Intubation, normal | 24 | 7.062 ± 56.836 | 758.694 | 0.526 |
|  | Intubation, difficult | 4 | 0.010 ± 0.011 | 0.048 | **0.018** |
|  | Extubation, normal tube | 19 | 1.779 ± 10.380 | 90.270 | 0.918 |
|  | Extubation, laser tube | 5 | 3.777 ± 10.330 | 29.334 | 0.774 |
|  | Extubation, non-coughing | 13 | 0.938 ± 4.652 | 29.334 | 0.794 |
|  | Extubation, coughing | 11 | 2.857 ± 13.457 | 90.270 | 0.903 |
|  | *Backgroundᵃ* |  | *0.005 ± 0.018* | *0.228* |  |
|  | *Coughingᵇ* |  | *1.588 ± 13.751* | *195.51* |  |
| 1-5𝜇m particle concentration, particles/cm³ | Intubation, normal | 24 | 0.017 ± 0.176 | 3.534 | 0.085 |
|  | Intubation, difficult | 4 | 0.002 ± 0.005 | 0.030 | **0.012** |
|  | Extubation, normal tube | 19 | 0.011 ± 0.014 | 0.078 | 0.310 |
|  | Extubation, laser tube | 5 | 0.008 ± 0.009 | 0.024 | 0.505 |
|  | Extubation, non-coughing | 13 | 0.005 ± 0.008 | 0.030 | 0.094 |
|  | Extubation, coughing | 11 | 0.015 ± 0.016 | 0.078 | 0.879 |
|  | *Backgroundᵃ* |  | *0.000 ± 0.002* | *0.018* |  |
|  | *Coughingᵇ* |  | *0.012 ± 0.064* | *1.242* |  |
| >5𝜇m particle concentration, particles/ cm³ | Intubation, normal | 24 | 0.001 ± 0.003 | 0.012 | 0.173 |
|  | Intubation, difficult | 4 | 0.001 ± 0.003 | 0.018 | 0.414 |
|  | Extubation, normal tube | 19 | 0.003 ± 0.005 | 0.030 | 0.138 |
|  | Extubation, laser tube | 5 | 0.001 ± 0.002 | 0.006 | 0.450 |
|  | Extubation, non-coughing | 13 | 0.002 ± 0.003 | 0.006 | 0.955 |
|  | Extubation, coughing | 11 | 0.004 ± 0.006 | 0.030 | 0.116 |
|  | *Backgroundᵃ* |  | *0.000 ± 0.001* | *0.006* |  |
|  | *Coughingᵇ* |  | *0.001 ± 0.002* | *0.012* |  |

Mean ± SD and maximum concentrations were calculated from all measured time points (every 10s.) for each procedure subcategory. Measured minimum value in all anaesthesia procedures and reference measurements in all size groups of particles was 0.000. *P*-values compared with aerosol concentrations of the background were calculated using one-tailed paired t-test, for coughing reference two-tailed unpaired t-test was used. *P-*values <0.05 were considered statistically significant. Mean difference and 95 % CI are presented in Supplemental Table 4.*ᵃ* Background reference: Values presented as combination of all measured operation room background values. *ᵇ* Coughing reference. *Number of patients. SD, standard deviation.
